# A case of pulmonary pleomorphic carcinoma associated with cystic airspace

**DOI:** 10.1016/j.radcr.2023.05.022

**Published:** 2023-05-31

**Authors:** Mamiko Iwamura, Miki Nishimori, Hitomi Iwasa, Michimi Otani, Kosuke Nakaji, Noriko Nitta, Kana Miyatake, Rika Yoshimatsu, Tomoaki Yamanishi, Tomohiro Matsumoto, Mitsuko Iguchi, Hironobu Okada, Takuji Yamagami

**Affiliations:** a Department of Diagnostic and Interventional Radiology, Kochi Medical School, Kochi University, Kohasu, Oko-cho, Nankoku, Kochi, 783-8505, Japan; b Department of Diagnostic Pathology, Kochi Medical School, Kochi University, Kohasu, Oko-cho, Nankoku, Kochi, Japan; c Department of Thoracic Surgery, Kochi Medical School, Kochi University, Kohasu, Oko-cho, Nankoku, Kochi, Japan

**Keywords:** Pulmonary pleomorphic carcinoma, Non-small cell lung cancer, Lung cancer associated cystic airspace

## Abstract

Lung cancer associated with a cystic airspace is frequently misdiagnosed or overlooked. Adenocarcinoma, followed by squamous cell carcinoma, is the most typical histologic type of lung cancer connected to a cystic airspace. Here we present the rare case of lung pleomorphic carcinoma associated with a cystic airspace. We encountered a 74-year-old Japanese man diagnosed by computed tomography (CT) as having a nodule outside a cystic airspace in the lung. Several previous CT images showed that the cystic airspace preceded the nodule. Postsurgery, pathology indicated a diagnosis of pleomorphic carcinoma. Since pulmonary pleomorphic carcinomas pursue an aggressive clinical course, their early detection may contribute to an improved prognosis. Our case demonstrated that pleomorphic carcinoma can arise with cystic airspaces. For early diagnosis of those aggressive lung cancers, chest physicians should carefully examine the walls of cystic airspaces on CT.

## Introduction

Pleomorphic carcinoma is rare [1], tending to occur in elderly male smokers [2]. This subtype of lung cancer has an aggressive clinical course and poor prognosis [3], [4], [5]. Recently, early lung cancers associated with cystic airspaces are increasingly being recognized as a cause of delayed diagnoses. Adenocarcinoma and squamous cell carcinoma are the most typical histologic types of lung cancer associated with cystic airspaces. However, pleomorphic carcinomas developing in cystic airspaces are rare. We herein present the case of lung pleomorphic carcinoma associated with a cystic airspace.

## Case presentation

The patient, a 74-year-old Japanese asymptomatic man, had been smoking 20 cigarettes a day beginning at the age of 20 years and up to the present. He underwent surgery for ascending colon cancer (stage I) 5 years previously at our digestive surgery department. His progress was favorable after the surgery. However, 5 years later, a nodule in the peripheral zone of the left lower lung was noticed during a postoperative follow-up for the ascending colon cancer. Nonenhanced computed tomography (CT) showed that a solid nodule (10 mm) protruded externally from a thin-walled cyst and touched the visceral pleura. The nodule had homogeneous density on the soft tissue window. Fluorodeoxyglucose positron emission tomography/computed tomography (FDG-PET/CT) revealed fluorodeoxyglucose (FDG) accumulation within the nodule (standard uptake value [SUV] max, 4.8; fasting blood glucose upon examination, 173 mg/dL). There were no lymph node or distant metastases. Tumor marker carcinoembryonic antigen (CEA) was increased at 7.7 ng/mL. The patient was referred from our digestive surgery department to our thoracic surgery department for further examination and treatment.

**Fig. 1:**
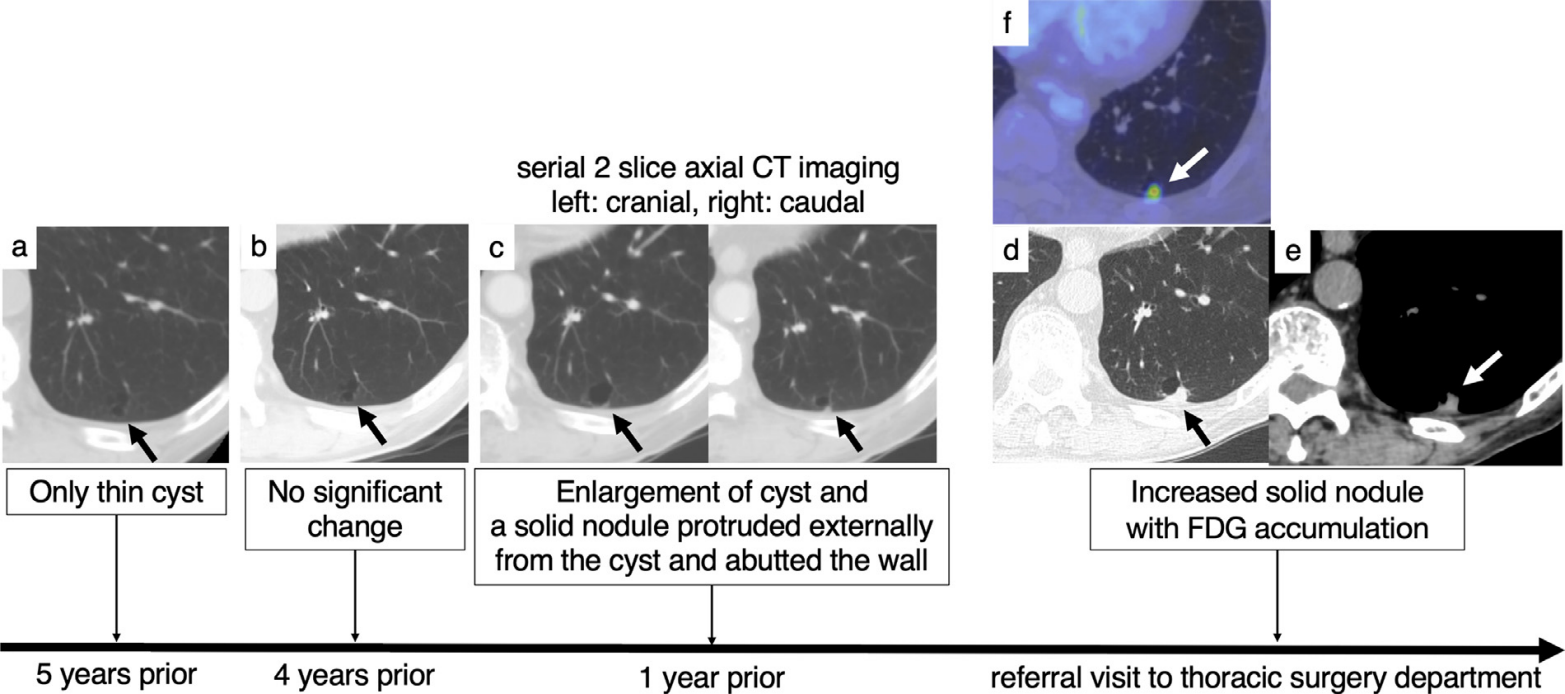
Disease evolution and correlation with imaging. (A) Axial CT scan obtained 5 years prior to the referral to our thoracic surgery department shows a left lower lobe thin-walled cyst (arrow). (B) Four years prior, the cyst did not significantly change. (C) Serial 2 slice axial CT (left: cranial, right: caudal) scan obtained 1 year prior. The cyst had enlarged and a solid nodule about 3 mm protruded externally from the cyst and abutted the wall (arrow). (D) Axial CT scan at the initial visit to our thoracic surgery department showed a thin-walled cyst and solid nodule that had increased to 10 mm (arrow). (E) Axial CT scan on soft tissue window showed homogeneous density of the nodule (arrows). (F) FDG-PET/CT revealed FDG accumulation within the nodule (arrow).

At the thoracic surgery department, previous CTs were reexamined. Five years (at surgery for the ascending colon cancer) and 4 years prior to the referral to our thoracic surgery department, only a thin-walled cyst could be seen on CT. However, 1 year prior to the referral, the cyst had enlarged and a solid nodule about 3 mm protruded externally from the cyst and abutted the wall. Fig. 1 shows the series of CT and FDG-PET/CT studies over the 5 years prior to the referral to our department of thoracic surgery.

**Fig. 2:**
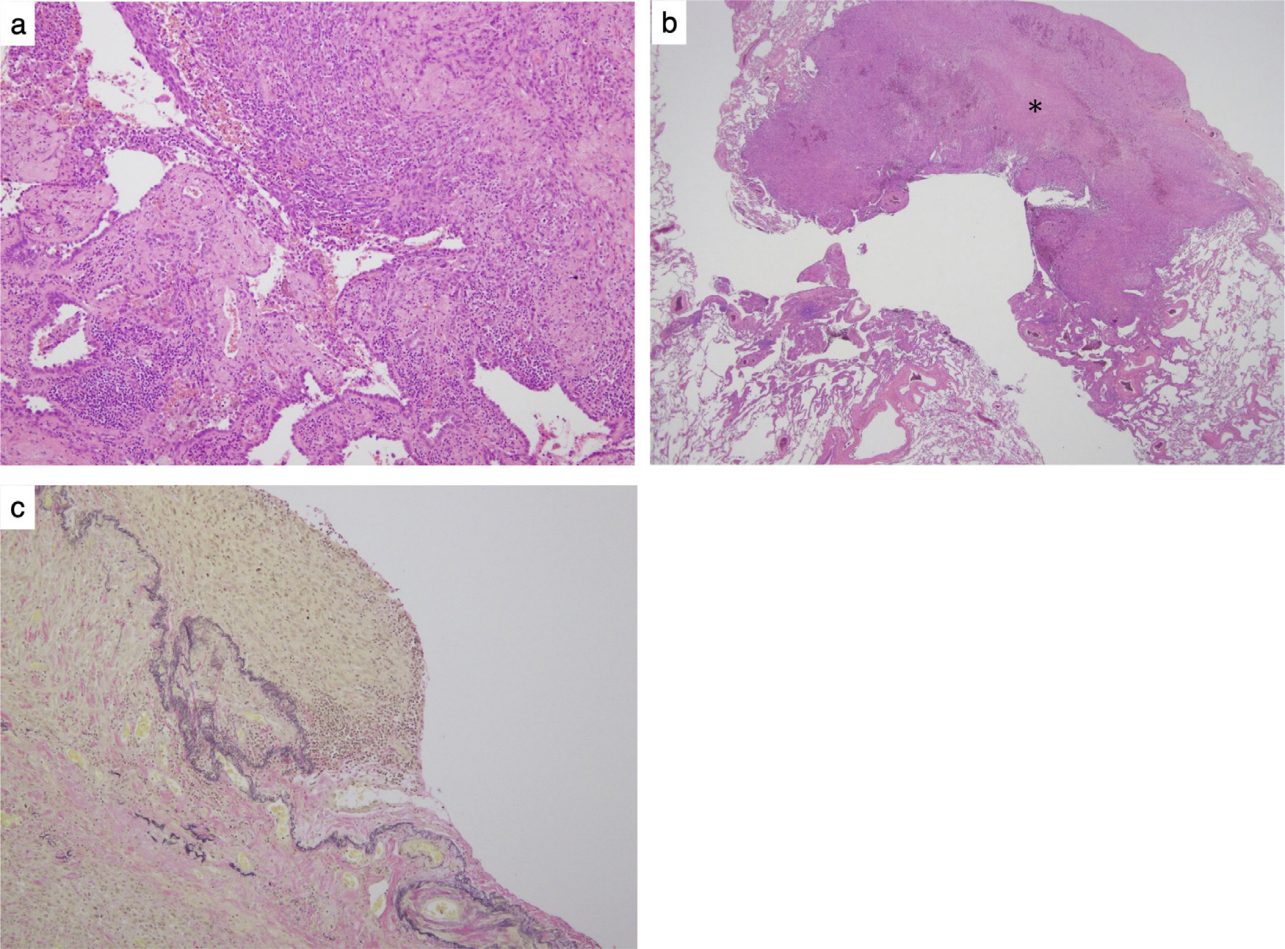
(A) Postoperative section with hematoxylin and eosin staining. The tumor is composed of an area of adenocarcinoma and a sarcomatoid component with mainly spindle-shaped cells. (B) Hematoxylin and eosin staining. The tumor included an area of necrosis (asterisk). (C) Elastica van Gieson (EVG) staining. Part of the tumor has infiltrated beyond the pleural elastic plate, slightly invading the visceral pleural surface.

As the previous ascending colon cancer was stage I and had not recurred during the 5 years after colon cancer surgery, there was little chance that the pulmonary nodule was a metastasis of the previous ascending colon cancer. These findings suggested a primary lung cancer associated with cystic spaces. After surgery, a pleomorphic carcinoma (mixture of adenocarcinoma and spindle cell carcinoma, pT2aN0cM0 Stage IB) was pathologically diagnosed (Fig. 2). The tumor included an area of necrosis. Elastica van Gieson staining showed that part of the tumor had infiltrated beyond the pleural elastic plate, slightly invading the visceral pleural surface. There were no pathologically suspicious results indicating metastatic colon cancer.

According to the patient's wishes, no adjuvant therapy was administered. Two years after surgery, there has been no recurrence of cancer.

## Discussion

Pleomorphic carcinoma is a rare subtype of sarcomatoid carcinoma [1]. Histologically, pleomorphic carcinoma is a non-small cell lung carcinoma that contains at least 10% spindle and/or giant cells or a carcinoma consisting only of spindle and giant cells [6]. These tumors occur mainly in men who smoke heavily [2]; the average age of onset is 65 years [7]. Clinical symptoms are generally nonspecific, such as cough, hemoptysis, fever, dyspnea, and chest pain, whereas some patients are asymptomatic [7,8]. These tumors pursue an aggressive clinical course, and the 5-year survival rate indicates a strong malignant potential and poor prognosis [3], [4], [5]. Tumor stage is an important prognostic factor; therefore, an early diagnosis is of vital importance to improve the prognosis for pleomorphic carcinoma.

Most pleomorphic carcinomas feature an epithelial component consisting of adenocarcinoma, squamous cell carcinoma, adenosquamous carcinoma, or large-cell carcinoma. Therefore, their radiological characteristics are frequently comparable to those of conventional nonsmall cell lung carcinoma. On CT imaging, pulmonary pleomorphic carcinoma sometimes presents as a large solitary mass, with tumors reported to range from 1.1 to 12.0 cm (mean 5.4 cm) [9]. Similar to our case, the tumor generally presents as a peripheral lesion and touches the visceral pleura widely on the preoperative chest CT. Furthermore, the tumor frequently invades the chest wall. Chen et al. [2] reported that patients with tumors invading the visceral pleural surface had significantly worse overall survival than patients without such invasive tumors. Moreover, among other prognostic factors, histologically diagnosed massive coagulation necrosis was reported [3]. Nishida et al. [9] reported that on CT imaging most tumors showed heterogeneous enhancement and contained a low-density area. Because preoperative enhanced CT had not been obtained in our case, the presence of necrosis in the tumor had not been recognized. However, in pathological specimens an area of necrosis was confirmed by pathologists despite the tumor's tiny size. Contrast-enhanced CT could have provided additional information about the tumor.

In this case, a pleomorphic carcinoma had developed in cystic airspaces, and this type of pleomorphic carcinoma is rare. The term “cystic airspace” includes congenital cysts, emphysematous bullae, fibrotic cysts, bronchiectatic airways, subpleural blebs, and cystic dilatation of distal airways arising de novo from small cancers owing to obstruction [10]. Adenocarcinoma, followed by squamous cell carcinoma, is the most typical histologic type of lung cancer connected to a cystic airspace according to Fintleman et al. [11]. Their pathologic analysis revealed that lung cancers with cystic airspaces correspond to a check-valve mechanism, adenocarcinoma superimposed on emphysema, cystification, and adenocarcinoma parasitizing a preexisting bulla. According to a previous report [12], the cystic wall is composed of Attenuated and compressed parenchyma and connective tissue, and microorganisms deposit on the wall because of restricted air flow in that region, resulting in recurring infections. Repeated inflammatory processes may result in the formation of a fibrous scar around the cyst and may lead to accumulation of carcinogens. However, research is currently underway to determine the exact mechanism of lung cancer carcinogenesis linked to cystic airspaces.

Lung cancers associated with a cystic airspace have been found in all lobes. Additionally, most of those tumors were peripheral or subpleural rather than central, in agreement with our case [13]. Regarding CT findings and classification, Maki et al. [14] were the first to describe a classification system for lung cancers associated with cystic airspaces. Mascalchi et al. [15] later modified this system. They classified lung cancer associated with cystic airspaces into 4 morphologic patterns: solid nodule protruding externally (type I) or internally (type II) from the cyst wall; circumferential thickening of the cyst wall (type III); and tissue intermixed within clusters of cysts (type IV). According to this classification, our case was classified as type I. Morphological findings and changes during follow-up in type I can be subtle and the lesions may become more complex [16]. Later, Shen et al. [17] classified these lesions into 4 types: type I, mean wall thickness <2 mm; type II, mean wall thickness ≥2 mm; type III: a cystic airspace with a mural nodule; and type IV, tissue intermixed within clusters of cystic airspaces. They reported that employing a multivariate analysis, lung adenocarcinoma with the type III morphological pattern was an independent risk factor for high pathological invasiveness. Our case highlights that, with the exception of highly invasive lung adenocarcinoma, pleomorphic carcinoma can also occur in a cystic airspace with a mural nodule. Based on our experience, careful observation of changes in the morphological features of a cystic airspace, especially in the presence of a mural nodule either endophytic or exophytic, is needed with thin-section CT images.

A study by Fintelmann et al. [11] showed that some cystic airspaces appeared in previously normal lungs, whereas others were preceded by nodules. In our case, a previous CT scan showed that the cystic airspace preceded the nodule. Mascalchi et al. [15] reported that cystic airspaces sometimes increased in size along with tumor growth, similar to our case, presumably reflecting a valve mechanism on the distal airway connected with the cystic airspace. Usually, a further increase in tumor diameter leads to a reduction in the size of the cystic airspace component. Furthermore, in some tumors the cystic airspace no longer remains. Therefore, early diagnosis before the replacement of the air space with a solid tumor is crucial.

Lung cancers associated with a cystic airspace have been increasingly identified by routine CT imaging and lung cancer screening programs. However, this type of lung cancer is frequently misdiagnosed or overlooked because of its rare morphology or that many other conditions (infection, inflammation, and granulomatous diseases, etc.) mimic it [10,18]. At present, unfortunately, there are no standardized management protocols for follow-up of these lesions because of their relative infrequency. Therefore, all physicians who specialize in thoracic oncology must be able to recognize and quickly diagnose lung cancer that is linked to cystic airspaces.

## Conclusion

Our case demonstrated that pleomorphic carcinoma, considered to have a poor prognosis, can arise with cystic airspaces. For early diagnosis of those lung cancers, chest physicians should carefully examine the walls of cystic airspaces on CT.

## Patient consent

Written informed consent for the publication of this case report was obtained from the patient.

## Ethics committee approval

This is a case report involving 1 patient; thus, institutional ethics committee approval was not required.
